# RNA-Seq Profiling to Investigate the Mechanism of Qishen Granules on Regulating Mitochondrial Energy Metabolism of Heart Failure in Rats

**DOI:** 10.1155/2021/5779307

**Published:** 2021-12-31

**Authors:** Hao He, Changxiang Li, Xiangyu Lu, Yanqin Li, Xuan Li, Xiaoqian Sun, Binghua Tang, Yan Wu, Chun Li

**Affiliations:** ^1^School of Life Science, Beijing University of Chinese Medicine, Beijing 100029, China; ^2^College of Traditional Chinese Medicine, Beijing University of Chinese Medicine, Beijing 100029, China; ^3^Modern Research Center of Traditional Chinese Medicine, School of Traditional Chinese Material Medica, Beijing University of Chinese Medicine, Beijing 100029, China; ^4^Beijing Key Laboratory of TCM Syndrome and Formula, Beijing, China; ^5^Key Laboratory of TCM Syndrome and Formula (Beijing University of Chinese Medicine), Ministry of Education, Beijing, China

## Abstract

*Background*. Qishen granules (QSG) are a frequently prescribed formula with cardioprotective properties prescribed to HF for many years. RNA-seq profiling revealed that regulation on cardiac mitochondrial energy metabolism is the main therapeutic effect. However, the underlying mechanism is still unknown. In this study, we explored the effects of QSG on regulating mitochondrial energy metabolism and oxidative stress through the PGC-1*α*/NRF1/TFAM signaling pathway. RNA-seq technology revealed that QSG significantly changed the differential gene expression of mitochondrial dysfunction in myocardial ischemic tissue. The mechanism was verified through the left anterior descending artery- (LAD-) induced HF rat model and oxygen glucose deprivation/recovery- (OGD/R-) established H9C2 induction model both *in vivo* and *in vitro*. Echocardiography and HE staining showed that QSG could effectively improve the cardiac function of rats with myocardial infarction in functionality and structure. Furthermore, transcriptomics revealed QSG could significantly regulate mitochondrial dysfunction-related proteins at the transcriptome level. The results of electron microscopy and immunofluorescence proved that the mitochondrial morphology, mitochondrial membrane structural integrity, and myocardial oxidative stress damage can be effectively improved after QSG treatment. Mechanism studies showed that QSG increased the expression level of mitochondrial biogenesis factor PGC-1*α*/NRF1/TFAM protein and regulated the balance of mitochondrial fusion/fission protein expression. QSG could regulate mitochondrial dysfunction in ischemia heart tissue to protect cardiac function and structure in HF rats. The likely mechanism is the adjustment of PGC-1*α*/NRF1/TFAM pathway to alleviate oxidative stress in myocardial cells. Therefore, PGC-1*α* may be a potential therapeutic target for improving mitochondrial dysfunction in HF.

## 1. Introduction

Cardiovascular disease has always been one of the major diseases threatening human health with the highest morbidity and mortality and remains one of the leading causes of death in the world [1]. Chronic heart failure (CHF) is caused by ventricular remodeling, myocardial energy metabolism disorder, neurohumoral regulation, and other pathways [2]. Mitochondrial dysfunction-induced cardiomyocyte apoptosis plays a dominant role in CHF [2, 3]. It is increasingly recognized that mitochondrion is an important organelle for the energy supply and includes important biological and regulatory roles such as redox balance, biosynthesis, reactive oxygen species (ROS) signaling, cell growth and death, ion homeostasis, protein quality control, and inflammation [4]. Mitochondrial function is also regulated by biogenesis, fission, fusion dynamics, and protein quality control via autophagy [5]. Therefore, early control of mitochondrial quality, reduction of mitochondrial dysfunction, and oxidative stress have become new targets for the treatment of cardiovascular diseases.

Peroxisome proliferator-activated receptor-*γ* coactivator *α* (PGC-1*α*) is regarded as the dominant coordinating factor involved in regulating mitochondrial metabolism and biogenesis [6]. During the growth of mitochondria, PGC-1*α* regulates mitochondrial function through the interaction with mitochondrial transcription factor A (TFAM) and nuclear respiratory factors (NRFs), which are a class of transcription factors that regulates the expression of genes in the mitochondrial respiratory chain [7, 8]. There are two main subtypes, nuclear respiratory factor 1(NRF-1) and NRF-2 [9]. By binding with NRF1/2, PGC-1*α* acted on the promoter of TFAM, thereby promoting mitochondrial DNA replication and transcription, regulating mitochondrial biosynthesis, and stimulating mitochondrial proliferation [10]. In addition, the homeostasis of TFAM plays a vital role in maintaining the normal electron transfer of the intracellular respiratory chain, which ensures the stability of cell energy metabolism [11, 12].

In China, traditional Chinese medicine (TCM) has been widely used to treat cardiac disorders for thousands of years. Numerous studies have revealed that TCM had important activity in heart failure [13–15]. Qishen granule (QSG), a Chinese herbal compound composed of *A. membranaceus* Bunge., *S. miltiorrhiza* Bunge., *L. japonica* Thunb., *S. aestivalis* Griseb., *A. fischeri* Rchb., and *G. uralensis* Fisch, is developed from an empirical formula of the classic prescription Zhenwu Tang and Si Miao Yong An Tang in TCM [16, 17]. Previous studies have shown that QSG could effectively improve cardiac function and energy metabolism in rats with heart failure while the potential pharmacological mechanism remains to be further defined [18–20]. Due to the complex components and effects of TCM compounds, we established the LAD-induced HF rat model to analyze the differentially expressed genes of QSG-treated rats by RNA-seq technique. Transcriptomics is a convincing method that offers reliable access to changes in the expression of genes, which could bring to light the involved mechanisms of drugs [21, 22]. Meanwhile, results from transcriptomics studies usually only indicate multiple pathways induced by the traditional Chinese medicine formula; however, relative contribution and emphasis of these pathways to their beneficial effects remain to be further verified by other pharmacological methods [22]. In this study, we have performed transcriptomics analysis and pharmacological experiments to clarify whether QSG regulates mitochondrial dysfunction and oxidative stress through PGC-1*α*/NRF1/TFAM signaling pathway and to lay a theoretical foundation for clinical QSG drug efficacy research.

## 2. Materials and Methods

### 2.1. Animals and Treatment

Male Sprague-Dawley (SD) rats weighing 220–240 g were purchased from Beijing Vital River Laboratory Animal Technology (license no. SCXK 20160006, Beijing, China). Following being acclimated to their housing environments for 1 week, the rats were randomly assigned into sham group, model group, and QSG group using a computer-generated random number table.

Ligation surgery of left anterior descending coronary artery was employed to establish a rat model of CHF, as our previous study reported [17, 23]. All the rats were anesthetized with pentobarbital sodium 45 mg/kg intraperitoneally. Left thoracotomy was performed layer by layer between the third and fourth intercostal space. After the cardiac tissues were separated, left anterior descending coronary artery was ligated with a sterile suture 1 mm below the left atrium. Rats in sham group were manipulated in the same way with no actual ligation of left anterior descending coronary artery. The thorax was closed layer by layer. The sham operation was carried out only by a surgical incision and stitch up. At the same time, the QSG was administered (18.66 g/kg) to QSG-treated group for 21 days as previous study [23]. The sham and model groups were treated with normal saline (10 ml/kg).

The rats were sacrificed at 21 days after surgery. Serum and cardiac samples were harvested for different analyses. All the animal welfare and experimental procedures followed the guidelines and practices established by the National Institutes of Health guide for the care and use of laboratory animals and were approved by the Ethics Committee of Experimental Animals of Beijing University of Chinese Medicine (BUCM-3-2016040201-2003).

### 2.2. RNA Isolation, Library Construction, and Sequencing

Total RNA was isolated from the infarct border zone of cardiac tissues for each biological replicate using the RNAprep Pure Tissue Kit (Beijing Tiangen Biochemical Technology Co., Ltd.) following the manufacturer's protocol. Each sample was divided into two parts: one was used for total RNA sequencing while the other was used for real-time quantitative PCR analysis. The purification of RNA was performed and mRNAs were reverse transcribed into cDNA using oligo (dT) primers. The cDNA library was sequenced at the HiSeq 2500 using standard procedures to obtain matched end reads of 50 bp. Clean reads were obtained by removing reads containing adapters, reads containing poly-N, and low-quality reads from raw data. Employing CASAVA software, the images generated by the Illumina sequencers were converted into raw reads by base-calling. To secure the quality of the data for following analyses, the clean reads were obtained from the raw data with three criteria to filter out the rough raw reads: removing the reads with sequence adaptors, removing the low-quality reads with more than 50% QA ≤ 19 bases, and removing the reads with more than 50% “N” bases. The gene expression was calculated by RPKM (reads per kilo bases per million reads) [24].

### 2.3. Gene Ontology and KEGG Pathway Functional Analysis and Enrichment Analysis

Cytoscape 3.4.0 software and its internal software ClueGO 2.3.3 CluePedia 1.3.3 and BiNGO software were used to perform Gene Ontology (GO) and Kyoto Encyclopedia of Genes and Genomes (KEGG) functional analysis and enrichment analysis on the differentially expressed genes verified by qRT-PCR. GO enrichment analyses were used to assess molecular function, biological pathway, cell composition, and pathway analysis. KEGG pathway and DAVID were used to understand high-level function and gene interaction network of differential expressed genes.

### 2.4. Verification of Target Genes by Quantitative Real-Time PCR (qRT-PCR) Analysis

The overall RNA from myocardial tissues was extracted using TRIzol. Double-stranded cDNA was synthesized from total RNA with Revert Aid First Strand cDNA Synthesis Kit (Thermo Fisher Scientific, K1622, USA). Real-time PCR analysis was proceeded using SYBR Select Master MIX and primers sequences. qRT-PCR was performed using a C1000 Thermal Cycler PCR instrument (Bio-Rad, Hercules, CA, USA). The sequences of the primers (Genomics, Beijing, China) were as follows: rat *Nd1*, 5′-CAATGTCCAAGATGCCTCCG-3′ and 5′-GCCCTCTCTTATCGCCAGAT-3′; rat *Cytb*, 5′-GGTGCTCGAGTACCTCACTG-3′ and 5′-CTTGTTGAGCTCCTCGTCGT-3′.

### 2.5. Echocardiographic Assessment of Cardiac Functions

28 days after surgery, echocardiography was used to assess cardiac functions (Vevo 2100, Visual Sonics, Canada). Left ventricular end-diastolic diameter (LVED), left ventricular end-diastolic diameter (LVED; d), ejection fraction (EF), and fractional shortening (FS) were evaluated by echocardiography. A PST 65A sector scanner (8 MHz probe) was used to generate two-dimensional guided images at a frame rate of 300 to 500 frames/sec. FS was calculated using the following formula: FS [(LVEDD−LVESD)/LVEDD] × 100%. EF was calculated using the following formula: EF [(LVEDV−LVESV)/LVEDV] × 100%.

### 2.6. Histopathological Examination

The rat cardiac tissues were fixed in 4% paraformaldehyde and embedded in paraffin. The heart tissues were sectioned into a thickness of 5 *μ*m slides. Sections were deparaffinized by immersion in xylene and stained with hematoxylin according to the standard protocol. The sections were analyzed under light microscope to visualize the structure of cardiomyocytes. Images were visualized under an optical OLYMPUS microscope at 40× magnification.

### 2.7. Transmission Electron Microscopy

The cardiac tissues were fixed in electron microscope fixing solution at room temperature for 2 hours and then transferred to 4°C for storage. The cardiac tissues were immersed in 0.5% osmium tetroxide solution at 4°C for 20 min. Samples were dehydrated in a series of graded ethanol and propylene oxide, stained with uranyl acetate and lead citrate, and then embedded in Luveak 812 (Nacal Cai Tesque). The mitochondria were examined under a Hitachi 7100 transmission electron microscope (Olympus, Tokyo, Japan).

### 2.8. OGD/R Insult on H9C2 and Drug Administration

The rat embryonic ventricular cell line H9C2 was purchased from National Experimental Cell Resource Sharing Service Platform (Beijing, China). The H9C2 cells were incubated in high-glucose DMEM with 10% FBS, 1% penicillin, and streptomycin in a humidified incubator with 5% CO_2_ at 37°C. The H9C2 cells were randomly divided into four groups: (1) control group, (2) OGD/R-induced model group, (3) QSG group (34.38 *μ*g/mL), and (4) QSG + SR-18292 group. To mimic myocardial ischemia, OGD/R were used on the H9C2 cells. Briefly, the medium was replaced with DMEM without glucose. Then the H9C2 cells were subjected to the sealed container with an anaerobic chamber (Mitsubishi, Tokyo, Japan) for 8 hours to initiate the OGD insult. OGD was terminated by complete medium. After this time, cell cultures were incubated under normoxic condition at 37°C for 12 hours to mimic reoxygenation. The H9C2 cells with the above treatment were defined as the model group. The cocultures in the control group were incubated in complete medium without OGD/R intervention. The cultures in QSG group were treated with QSG in DMEM without glucose under OGD conditions for 8 h. Subsequently, the H9C2 cells were cultured in complete medium for 12 h under normal growth conditions. The administration route of the SR-18292 group was to add SR-18292 on the condition of QSG.

### 2.9. Measurement of Indicators by Western Blot

Proteins collected from myocardial tissues and H9C2 cells were lysed by RIPA lysate (Beijing Applygen Technolog Inc., China) according to the manufacturer's instruction. The protein content was quantified using the BCA method. After addition of loading buffer and boiling for 5 minutes at 99°C, protein samples (25 mg per sample) were separated by 10% Tris/Glycine SDS-PAGE and then electrotransferred to PVDF membranes at 300 mA for 1.5 h. Membranes were incubated in TBST containing 5% nonfat milk for 1 hours. Then the membranes were incubated with different primary antibodies including rabbit monoclonal antibodies against MFN1/2 (Proteintech), OPA1 (Proteintech), FIS1 (Proteintech), DRP1 (Proteintech), PGC-1*α* (Proteintech), NRF1(Proteintech), TFAM (Proteintech), and GAPDH (Proteintech) overnight at 4°C. The blots were washed with TBST three times and then incubated with anti-rabbit IgG (1 : 2000) for 4 h at 4°C. After washing three times with TBST, the immunolabeling was detected using Omni ECL reagent (EpiZyme, China) for 1 min at room temperature. The ultimate expression of every single protein was standardized by GAPDH. The band grayscale analysis was performed using Image J software.

### 2.10. Measurement of ATP

The contents of ATPnase (Nanjing Jiancheng Bioengineering Institute, China) in cardiac tissues and H9C2 cells in different groups were assessed using kits according to the manufacturer's instructions. The optical density of samples was detected at the wavelength of 450 nm using a microplate reader (PerkinElmer, Waltham, MA, USA).

### 2.11. Measurement of JC-1

To evaluate the ΔΨm H9C2 cells, we applied the JC-1 staining to quantify the mitochondrial membrane potential. H9C2 cells in different groups were harvested and washed three times with PBS. After respective operations, cells were added to fresh medium containing the JC-1 (Beyotime Biotechnology, China) molecular probe (1 mL) and cultured at 37°C for 30 min. The H9C2 cells were then washed three times with PBS and JC-1 aggregates (red fluorescence) and JC-1 monomers (green fluorescence) were visualized under Olympus fluorescence microscopy at 40 × magnification.

### 2.12. Measurement of Oxidative Stress (ROS, GSH-PX, and iNOS)

H9C2 cells in different groups were washed with PBS and ROS produced by cells was measured by oxidant sensitive probe. DCFH-DA (10 *μ*M) was added to fresh medium and incubated at 37°C for 30 min. The H9C2 cells were then washed three times with PBS and were visualized under the Olympus fluorescence microscopy at 40 × magnification. GSH-PX (Jiancheng, Nanjing, China) and iNOS (Jiancheng, Nanjing, China) were measured using commercial kits.

### 2.13. Statistics

All experiments were made in triplicate or more. All data were expressed as mean ± standard deviation. A one-way analysis of variance (ANOVA) was used for multiple comparisons and SPSS 20.0 (SPSS, Chicago, IL, USA) or GraphPad Prism 7 was used for the least significant difference (LSD) test for each set of data. The values of *P* < 0.05 were considered statistically significant, while a difference of *P* < 0.01 was highly significant difference.

## 3. Results

### 3.1. RNA-Seq and qRT-PCR Showed That QSG Could Improve Heart Function in HF Rats, Which Was Closely Related to the Regulation of Mitochondrial Function

**Figure 1:**
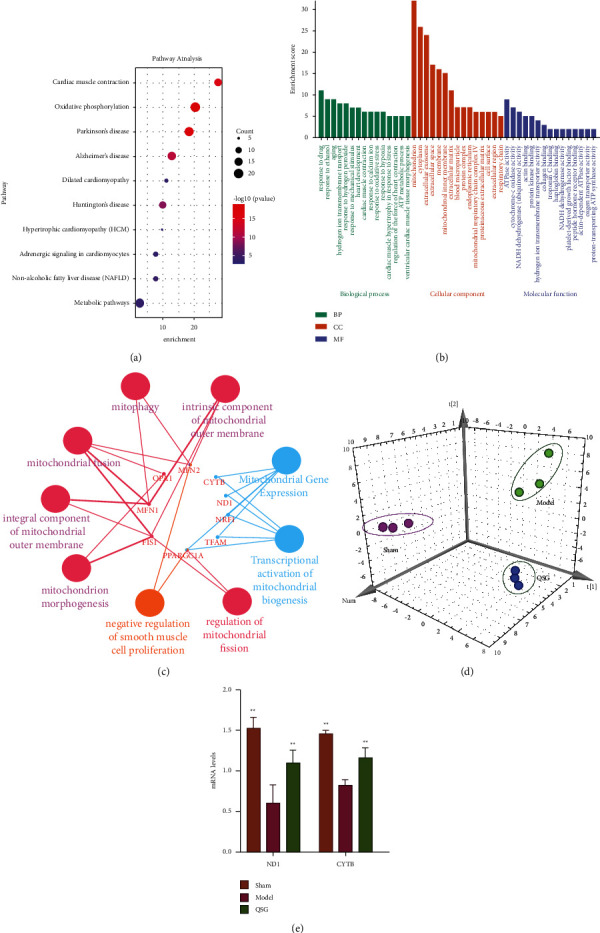
RNA-seq proved that QSG improved heart function of HF rats by regulating mitochondrial function. (a) KEGG pathways enrichment analysis of DEGs compared with MODEL and QSG. (b) GO annotation enrichment analysis of DEGs compared with MODEL and QSG. (c) PPI protein interaction analysis and for differential genes. (d) OPLS-DA 3D diagram of sham operation group (sham), model group (model), and Qishen granule group (QSG). (e) Effects of QSG on the mRNA expressions of the significantly altered genes in MI rat heart tissues by qRT-PCR assay. The expression of each target was normalized to that of the GAPDH. ^*∗*^*P* < 0.05, ^*∗∗*^*P* < 0.01, *n* = 3.

To investigate the transcriptomic responses associated with QSG treatment in HF, we performed RNA-seq on cardiac ischemic tissues. The RNA-seq analysis identified 52 upregulated and 40 downregulated genes associated with QSG treatment out of the 32,547 genes mapped to the database. Furthermore, we performed GO biological process analysis, KEGG pathway enrichment analysis, PPI protein interaction analysis, and so on for differential genes, combined with qRT-PCR experiments to verify the reliability of transcriptomics results. The enrichment results of KEGG pathway suggested that the drug target of QSG may be related to cell energy metabolism (Figure 1(a)). The GO results showed that the differential gene function mainly involved ATPnase activity, cytochrome C oxidase activity, NADH dehydrogenase/ubiquinone activity, actin binding, protease binding, and other classical biological processes (Figure 1(b)). Such functions were highly related to mitochondrial functional metabolism and energy metabolism (Figure 1(c)). The OPLS-DA diagram showed that the sequencing data of sham group, model group, and QSG group were completely separated. The difference between groups was obvious and the data was reliable (Figure 1(d)). In particular, QSG can significantly recall the mitochondrial coding genes *Cytb* and *Nd1*. *Nd1* is the main member of the cell's electron transport chain, which is located on the inner mitochondrial membrane and is responsible for pumping out protons. *Cytb* is part of the mitochondrial respiratory chain, which helps to generate a proton gradient across the mitochondrial membrane and is used for ATP synthesis. At the same time, the qRT-PCR results proved that the change trend of differential genes was consistent with the RNA-seq results and QSG could significantly adjust the expression of *Cytb* and *Nd1* (Figure 1(e)). It was suggested that QSG can improve mitochondrial dysfunction in rats with heart failure. Mitochondrial dysfunction is caused by a variety of abnormal biological processes, of which abnormal mitochondrial biosynthesis plays an important role. Current research shows that PGC-1*α* is a key regulator of mitonuclear communication, which may interact with nuclear factor, nuclear respiratory factor 1/2 to manage mitochondrial biogenesis [25]. However, whether QSG regulated mitochondrial biogenesis and improved mitochondrial dysfunction through PGC-1*α* was still unknown. Therefore, we further proved the above mechanism through *in vitro* and *in vivo* experiments.

### 3.2. QSG Can Effectively Improve Heart Function and Mitochondrial Structure Damage of HF Rats

**Figure 2:**
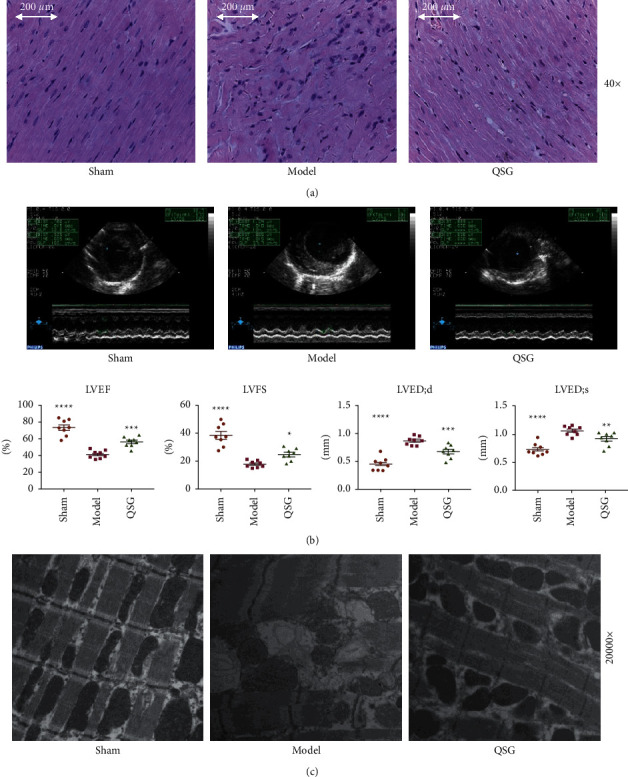
The effect of QSG on the heart function and mitochondrial structure of HF SD rats after AMI. (a) Myocardial samples of the three groups surrounding an area of infarction visualized via H&E staining and (40×). (b) Bar graphs of LV parameters (EF, FS, LVEDD and LVEDS). *n* = 8 per group. Values are mean ± SE. Asterisks indicate significant differences. ^*∗∗*^*P* < 0.01. (c) Effects of QSG on mitochondrial morphology in HF SD rats after LAD. (c) Effects of QSG on mitochondrial morphology in HF SD rats after LAD. ^*∗*^*P* < 0.05, ^*∗∗*^*P* < 0.01, ^*∗∗∗*^*P* < 0.001, *n* = 3.

Firstly, we proved that QSG can improve the heart function of HF rats. H&E staining displayed that cardiomyocytes in the sham group were orderly arranged. The nucleus of cardiomyocytes is mostly located in the middle of the cell, with an oval or rectangular shape. The pathological findings of myocardial tissue in the model group were obviously abnormal with unclear cell outline, pyknotic dark-staining, irregular nuclei, and infiltration of a large number of inflammatory cells. QSG treatment fully attenuated ischemic induced histopathology damage in cardiac tissues (Figure 2(a)). The results exhibited that, compared with the model group, the cell outline and inflammatory cell infiltration of the QSG group were ameliorated.

Echocardiography showed that the EF and FS of the model group were lower than those of the sham group (*P* < 0.01, Figure 2(b)). Meanwhile, LVEDD and LVEDS in model group increased significantly. Changes of these indicators suggested serious impairment of cardiac function. However, treatment with QSG led to a decrease in LVEDD and LVEDS and an increase in EF and FS (Figure 2(b)) compared with the model group, indicating that QSG could improve cardiac functions in HF.

Further, through transmission electron microscope observation of QSG to alleviate mitochondrial morphological damage, in the sham group, the morphology and structure of the myocardial cells were normal, the muscle filaments were neatly arranged, Z-line was clear, the nuclear morphology was regular, the mitochondria were densely distributed between the muscle filaments, and the shape of the mitochondria was mostly oval. The structure of myocardium cells in model group was fuzzy and the myofilaments were arranged irregularly, accompanied by the rupture of myofilaments and vacuolar degeneration. Compared with the model group, the damaged morphology of mitochondria in the QSG group was significantly improved and the mitochondria were neatly arranged (Figure 2(c)).

### 3.3. QSG Alleviated Mitochondrial Dysfunction and Oxidative Stress Injury of Infected Myocardium

Oxidative stress is the main cause of mitochondrial dysfunction. Oxidative stress can inhibit the activity of mitochondrial respiratory enzymes, slow down the electron transfer of respiratory chain, lead to mitochondrial dysfunction, and then accumulate a large amount of reactive oxygen species in mitochondria. Excessive reactive oxygen species can change mitochondrial membrane permeability, lead to mitochondrial membrane potential disorder, damage mitochondrial function, and then cause serious cell damage.

**Figure 3:**
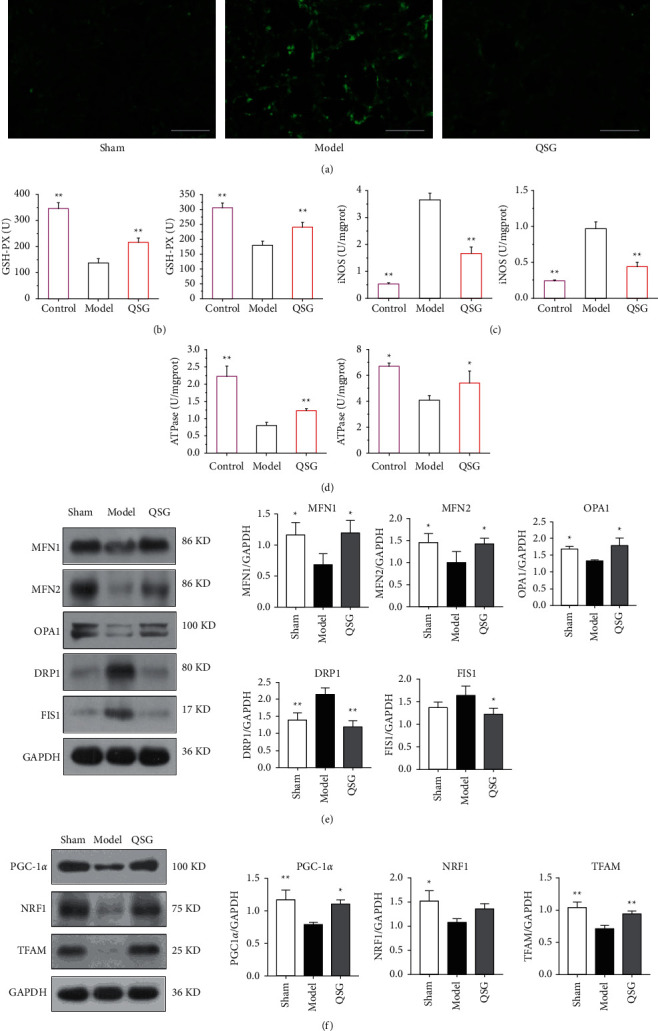
QSG regulated oxidative stress and mitochondrial biogenesis. (a) The level of ROS on H9C2 cells after OGD/R. (b) The level of GSH-PX on H9C2 cells after OGD/R. The level of GSH-PX on HF SD rats after AMI. (c) The level of iNOS on H9C2 cells after OGD/R. The level of iNOS on HF SD rats after AMI. (d) The level of ATPnase on H9C2 cells after OGD/R. The level of ATPnase on HF SD rats after HF. (e) QSG regulated the balance of mitochondrial fusion (e.g., MFN1/2, OPA1) and fission (DRP1, FIS1) in HF SD rats. (f) Effects of QSG on the expression of PGC-1*α*, NRF1, and TFAM in HF SD rats. ^*∗*^*P* < 0.05, ^*∗∗*^*P* < 0.01, *n* = 3.

As HF can induce prominent oxidative stress, we attempted to investigate whether QSG had antioxidative activity during HF. To this end, we used two models, the HF rat model and H9C2 cells exposed to OGD/R. QSG treatment effectively restored these levels in serum and cellular supernatant. QSG treatment effectively reduced the content of ROS (Figure 3(a)) and also restored the amount of glutathione PX (GSH-PX) (Figure 3(b)), iNOS (Figure 3(c)), and ATPnase in cardiomyocyte (Figure 3(d)).

In order to further verify the results of RNA sequencing, we used western blot to detect the expression levels of mitochondrial proteins (PGC-1*α*, TFAM, NRF1) and mitochondrial dynein (MFN1/2, OPA1). The results showed that PGC-1*α*, TFAM, NRF1, and mitochondrial fusion proteins MFN1/2, OPA1 attenuated in the model group, while the expression of mitochondrial fission proteins DRP1 and FIS1 increased in the model group. Furthermore, Figures 3(e) and 3(f) proved that QSG can effectively enhance PGC-1*α*, TFAM, NRF1, MFN1/2, and OPA1 and reduce the expression of DRP1 and FIS1 after administration. In summary, the results of western blot were basically the same as those of RNA sequencing.

### 3.4. QSG Regulated the Process of Mitochondrial Biogenesis of Myocardial Injury

Preliminary studies have proved that the expression of ROS in the H9C2 induced by OGD/R was significantly increased, which corresponds to the in vivo animal model. Therefore, we selected this cell model to verify whether QSG regulated the expression of mitochondrial fusion protein and fission protein in OGD/R-induced H9C2 cell model through the PGC-1*α*/NRF1/TFAM signal pathway.

**Figure 4:**
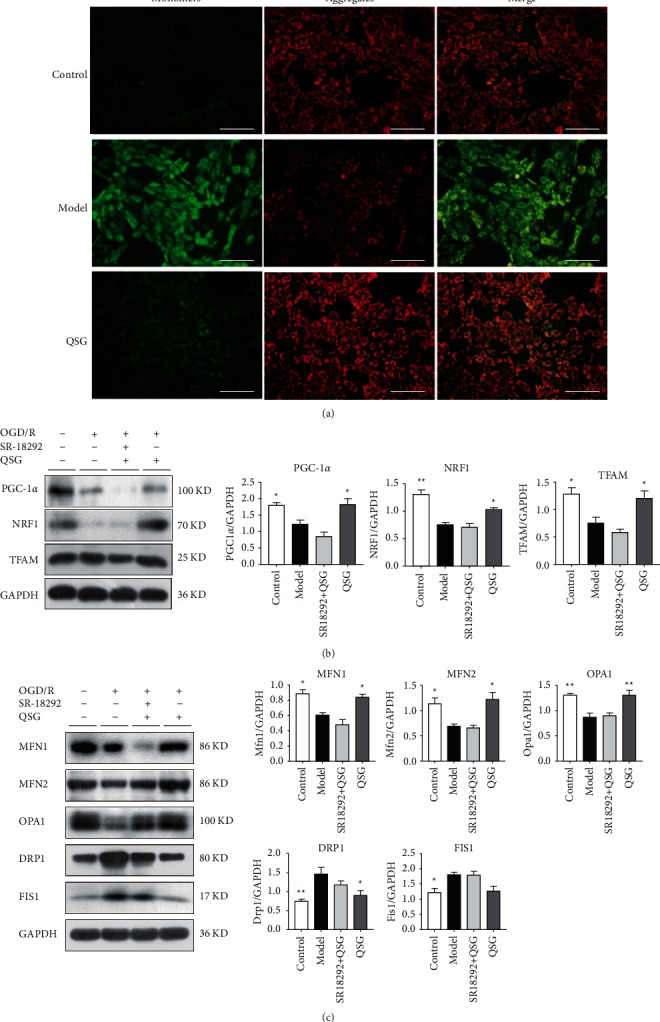
QSG through PGC-1*α*/NRF1/TFAM signaling pathway improved myocardial mitochondrial biogenesis. (a) Effects of QSG on JC-1 on H9C2 cells after OGD/R. (b) Effects of QSG on the expression of PGC-1*α*, NRF1, and TFAM on H9C2 cells after OGD/R. (c) QSG regulated the balance of mitochondrial fusion (e.g., MFN1/2, OPA1) and fission (DRP1) on H9C2 cells after OGD/R. ^*∗*^*P* < 0.05, ^*∗∗*^*P* < 0.01, *n* = 3.

First, we proved that QSG can regulate the mitochondrial transmembrane potential. The mitochondria of normal control group showed red fluorescence. However, the red fluorescence signal decreased and the green fluorescence signal increased significantly in the model group with OGD/R injury. JC-1 emits red fluorescence in high-energy mitochondria and green fluorescence in depolarized mitochondria. Therefore, the enhancement of red fluorescence and the decrease of green fluorescence in QSG group can prove that QSG can treat the injury caused by Δ*ψ* M-induced H/R injury (Figure 4(a)). The results suggested that QSG could maintain the structural integrity of mitochondrial membrane.

In order to further verify the mechanism of QSG in the treatment of HF, we evaluated the effect of QSG in the OGD/R cell model. Western blot showed that QSG upregulated the expression of PGC-1*α*, NRF1, and TRAM (Figure 4(b)). QSG treatment also activated the expression of mitochondrial fusion proteins, including MFN1, MFN2, and OPA1. In addition, after treatment with QSG, expression of mitochondrial fission proteins DRP1 and FIS1 was downregulated (Figure 4(c)). Finally, the PGC-1*α* inhibitor attenuated the therapeutic effect of QSG, proving that the regulation of mitochondrial function by QSG was mediated by PGC-1*α*.

## 4. Discussion

Heart, the energy consuming organ in the body, plays a remarkable role in energy metabolic homeostasis. Mitochondria account for 30% of the total volume of cardiomyocytes, which is the main place for energy production of cardiomyocytes and an important organelle that regulates myocardial function [26]. Mitochondria mainly regulate the biogenesis, fusion, fission, and autophagy of mitochondria to ensure the relative stability of mitochondrial morphology, quantity, and quality and maintain the integrity of its structure and function [27]. Studies reveal that myocardial ischemia and hypoxia can lead to mitochondrial dysfunction and oxidative stress and ultimately inhibit the physiological function of the myocardium [28–30]. Hence, regulating the key molecules in mitochondrial biogenesis could provide an alternative therapeutic approach for the treatment of heart failure.

We explored the effects of QSG on mitochondrial dysfunction and oxidative stress and discussed the mechanisms of mitochondrial biogenesis, fusion, and fission in this research. A rat model of post-AMI HF was established by left coronary vascular ligation [31]. Our findings indicated that QSG could significantly improve the cardiac function and pathological injury in the HF rats. Due to the complex components and effects of traditional Chinese medicine compounds, transcriptomics techniques were applied to obtain HF-related altered molecules and evaluate the therapeutic effects of QSG. RNA-seq technology analyzed gene expression in each group, and the DEGs were identified. The results demonstrated that myocardial infarction modified global gene expression with 38 upregulated and 29 downregulated genes in post-AMI HF. QSG treatment restored the normal expression of 27 of these genes.

KEGG pathway analysis further showed that oxidative phosphorylation and cardiac muscle contraction were the most significantly enriched pathways after QSG treatment. The results of KEGG and GO together suggested that QSG may play a cardioprotective effect by regulating energy metabolism and mitochondrial dysfunction. Energy metabolism has been proven to change during heart failure, so improving cardiac energy metabolism has been considered a potential method for the treatment of heart failure. Among them, *Nd1* and *Cytb* upregulated by QSG are mitochondrial-encoded genes which are an important part of mitochondrial respiratory chain complex I [32, 33]. The mitochondrial respiratory chain is located on the inner mitochondrial membrane, also called the electron transport chain. The chain is the main place where oxidative phosphorylation occurs. Nuclear respiratory factors (NRF1/2) are shown to act downstream of PGC-1*α* in driving the transcription and replication of the mitochondrial genome through activating mitochondrial transcription factor A (TFAM) [34, 35]. Studies have shown that PGC-1*α* is an important pathway for mitochondrial biogenesis [36, 37]. Hence, through these regulatory factors, nuclear DNA is involved in controlling the expression of mitochondrial DNA- (mtDNA-) encoded genes, like, seven subunits of NADH-dehydrogenase-ubiquinone reductase (ND1–ND6 and ND4L), cytochrome b (*Cytb*) of ubiquinol-cytochrome c reductase, three subunits of cytochrome c oxidase (CO I, CO II, and CO III), and two subunits of ATP synthetase (ATP6 and 8) involved in mitochondria OXPHOS [34, 38]. Under the action of PGC-1*α*, the capacity of mitochondrial DNA increased approximately, the mitochondria proliferate, and the density increased, and mitochondrial biosynthesis was activated. PGC-1*α* can activate the downstream targets NRF1 and NRF2, and then NRF1/2 binds to the nuclear mitochondrial transcription factor TFAM to regulate the replication and transcription of mtDNA [39–41], thereby promoting mitochondrial biosynthesis. In myocardial cells, the PGC-1*α*/NRF1/TFAM pathway is essential for regulating the mitochondrial function and antioxidant response [8]. Therefore, we investigated the assumption that QSG enhanced the mitochondrial function and antioxidant response via the pathway through *in vivo* and *in vitro* experiments.

**Figure 5:**
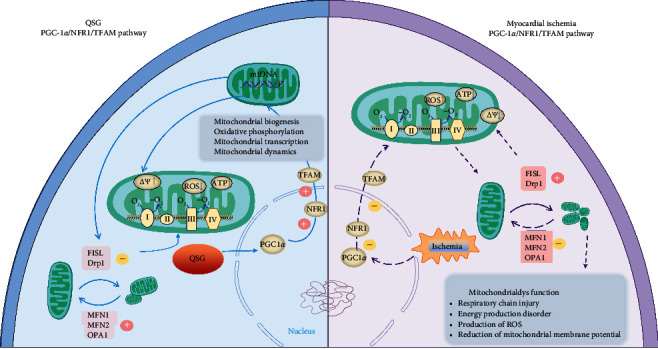
A schematic showing the cardioprotective effects of QSG by improving mitochondrial function through PGC-1*α* pathway.

In this study, both *in vivo* and *in vitro* experiments proved that the key factors of PGC-1*α*/NRF1/TFAM signaling pathway were reduced in rats with heart failure, and QSG can upregulate the expression levels of these key factors (Figure 5). It showed that QSG regulated mitochondrial biosynthesis by enhancing mitochondrial function. QSG could also act on the expression of mitochondrial fusion/split protein, upregulate the expression of MFN1/2 and OPA1, inhibit the expression of DRP1 and FIS1, and reduce mitochondrial disorders and oxidative stress damage caused by heart failure. In summary, it was suggested that QSG may play an indispensable role in regulating mitochondrial dysfunction through the PGC-1*α*/NRF1/TFAM signaling pathway. In addition, restoring myocardial mitochondrial biogenesis may be a promising strategy for the management of heart failure.

## 5. Conclusion

By applying transcriptomics technology, we identified mitochondrial dysfunction as a potential direction for QSG in HF treatment. We found that QSG could promote the mitochondrial function and antioxidant response via the PGC-1*α*/NRF1/TFAM pathway to alleviate the effects of myocardial ischemia on mitochondrial dysfunction and oxidative stress in myocardial cells. Therefore, PGC-1*α* may be a potential therapeutic target for improving mitochondrial dysfunction in myocardial cells.

## Data Availability

The data used to support the findings of this study are available from the corresponding author upon request.
